# A Systematic Review of the Relationship Between Maltreatment and Mentalization in Middle Childhood

**DOI:** 10.1007/s40653-026-00924-0

**Published:** 2026-06-06

**Authors:** Asa Kerr-Davis, Meryl Westlake, Nick Midgley, Saul Hillman

**Affiliations:** 1 Anna Freud, London, UK; 2 University College London (UCL), London, UK

**Keywords:** Maltreatment, Mentalization, Theory of mind, Middle childhood, Epistemic trust

## Abstract

Theorists have recently argued that difficulties with mentalization (also described as theory of mind) may be an important mechanism linking childhood maltreatment to poor mental health outcomes. This systematic review examined evidence of the relationship between maltreatment exposure and difficulties with mentalization during the understudied period of middle childhood. A systematic search of Web of Science Core Collection, Scopus, PsycInfo, MEDLINE, IBSS, and grey literature databases from 1st January 1980–22nd February 2024 was conducted (*n* = 8156). Articles were eligible for inclusion if: (1) exposure to child maltreatment was reported; (2) the sample was mean age 5–12 years; (3) a measure of mentalization and/or theory of mind was used. 13 articles were included. Although the quality of literature and variety of methodologies precluded meta-analyses, 12 of the studies reported evidence of poorer mentalization/ theory of mind among maltreated children. These provisional findings are discussed with consideration of recent theoretical developments.

**Supplementary Information:**

The online version contains supplementary material available at https://doi.org/10.1007/s40653-026-00924-0.

## Introduction

Despite considerable evidence linking experiences of maltreatment in childhood to neurobiological, physiological, psychological, and social outcomes across the lifespan (Baldwin et al., 2023; Bick & Nelson, 2016; Carr et al., 2020), the psychological mechanisms which explain these developmental consequences remain poorly understood (Coughlan et al. 2022; Fonagy et al. 2017b, 2019; McCrory et al. 2022). Several theories have proposed possible mechanisms, including emotional dysregulation; attention bias for emotional cues; and difficulties with social competence (Pollak, 2015). This review will focus on the mentalization-based developmental theory.

The mentalization-based theoretical framework suggests that the relationship between childhood maltreatment and subsequent transdiagnostic mental health difficulties may be mediated by ‘mentalization’ or ‘theory of mind’ – terms which refer to the capacity to think in terms of the mental states of others and oneself (Baron-Cohen et al., 1985; Chiesa & Fonagy, 2014; Fabiola et al., 2020; Fonagy, 2008; Fonagy & Allison, 2012; Taubner et al., 2016). This theory proposes that maltreatment causes difficulties with making sense of social information about mental states (i.e., mentalizing), and poor mentalizing leads in turn to mental health difficulties by undermining the capacity for resilient social functioning (Bora 2021; Fonagy et al. 2017a; McCrory et al. 2019; Sharp et al. 2011; Sprung et al. 2022). However, recent empirical research has shown mixed and inconclusive results about the mediational role of mentalization in linking maltreatment and adult mental health outcomes, and the foundational premise that maltreatment and mentalizing are directly associated has not yet been robustly demonstrated during childhood (Bateman et al., 2023; Yang & Huang, 2024). The present systematic review therefore examines the literature to explore whether there is evidence of a direct negative association between maltreatment and children’s capacity to mentalize in middle childhood.

The term ‘mentalization’ originates in theory of mind literature (Leslie, 1987), and the two terms continue to significantly overlap (Fonagy, 2022). Theory of mind research originally placed greater emphasis on the mental activity of representing others’ cognitive states such as thoughts and beliefs (Fonagy & Target, 1997; Wellman, 2018), whereas mentalization theory incorporated pre-conscious, implicit, and affective aspects of mental activity in relation to both self and others (Choi-Kain & Gunderson, 2008; Fonagy, 2008). This has led some researchers to view mentalization as a broad ‘umbrella’ concept in contrast to the narrower cognitive concept of theory of mind (Choi-Kain & Gunderson, 2008). However, some researchers have used theory of mind measures to test mentalization theory (Fonagy et al. 1997a, b), and others have incorporated affective and self-mentalizing into theory of mind research (Happé, 2003; Jarvis et al., 2023). As a result, the two concepts have substantially converged in usage, such that contemporary theorists have argued that they are now, essentially, synonymous (Fonagy, 2022). For parsimony, the present review will use the overarching term ‘mentalization’ to refer to both mentalization and theory of mind: the mental activity which enables humans to perceive and interpret behaviour in terms of intentional mental states such as needs, desire, feelings, beliefs, and goals (Duschinsky & Foster, 2021; Fonagy et al., 2002).

Children’s early interpersonal experiences, especially attachment relationships, are thought to be crucial in shaping the emerging ability to mentalize (Brink et al., 2015; Fonagy et al., 2014; Hughes et al., 2005; Symons & Clark, 2000). Mentalization theory proposes that attuned caregiver relationships, in which infants’ affective displays are noticed, mentalized, and responded to sensitively and contingently, enable children to learn about their own affective states and to gradually form a socially constituted sense of self (Camoirano, 2017; Fonagy et al., 2002; Gergely, 2007). Children who are accurately mentalized by their caregivers are more likely to develop secure attachments (Fonagy et al., 1991; Meins et al., 2002), which provide a foundation for their own balanced capacity to mentalize themselves and others (Adrian et al., 2005; Aldrich et al., 2021). Conversely, poor parental mentalizing is associated with infant attachment insecurity (Zeegers et al., 2017), and attachment insecurity is linked with poorer theory of mind abilities in childhood (Fonagy, Redfern et al., 1997).

Maltreatment may have a particularly disruptive effect on children’s developing capacity to mentalize (Pang et al., 2022; Terradas et al., 2021). Caregivers who maltreat their children may have a poorer capacity to mentalize their child, leading to an increased likelihood of responding to their children’s emotional signals with mis-attuned or frightening behaviour (Rosso, 2022). Consequently, maltreated children may experience the minds of others as hostile, unpredictable, or unsafe, and may develop a sense that mentalizing is an unsafe or unhelpful activity (Allen, 2019; Luyten & Fonagy, 2019; Shipman & Zeman, 1999). There is some empirical evidence of direct links between maltreatment and aspects of poorer mentalizing in young children (Pears & Fisher, 2005), adolescents (Borelli et al., 2015; Luce & Tremblay, 2010; Martin-Gagnon et al., 2023), and adults (Garon-Bissonnette et al., 2023; Peterson et al., 2022; Yang & Huang, 2024). However, it is important to establish whether these findings show developmental continuity across childhood, and especially during the socially complex period of middle childhood.

Maltreatment may have distinctive impacts on mentalization during middle childhood (ages 5–12 years), as more complex forms of mentalizing typically emerge during this developmental stage (Ensink & Mayes, 2010). The paradigmatic ‘false-belief’ task to assess theory of mind is typically passed at 4 years of age (Premack & Woodruff, 1978), but the period after this has received comparatively little research attention (Bosco et al., 2016; Devine & Lecce, 2021). In most Western cultures, children’s social worlds significantly expand during middle childhood as they spend more time in the primary school environment, coinciding with the development of increasingly elaborate linguistic, emotional, and cognitive capacities (Carr, 2017; Smogorzewska et al., 2020). Children in middle childhood typically develop elaborate representational capacities, enabling them to interpret ambiguous social information (Devine & Hughes, 2013) and apply this information to take others’ perspectives in more complex situations (Lagattuta et al., 2015). Since middle childhood is an important period in the development of mentalization, it is therefore necessary to examine whether maltreatment has specific impacts on mentalizing during this developmental stage.

Establishing evidence of the effects of maltreatment on mentalization depends on how this capacity is assessed. Children are typically aged 7–8 when they develop the ability to pass standard tests of ‘advanced’ second-order theory of mind, which assess whether a child can consider what one agent would think that another agent’s belief would be, based on the information that each agent has available to them (Miller, 2013; Perner & Wimmer, 1985). More complex third- and fourth-order theory of mind tasks are not passed until age 10–11 (Lagattuta et al., 2015; Liddle & Nettle, 2006), and some affective tasks can discriminate between emotion recognition capacity into adulthood (Baron-Cohen et al., 2001). Whilst several structured tasks assess theory of mind development (Fu et al., 2023; Osterhaus & Bosacki, 2022), fewer measures from the mentalization research tradition are designed for use with children (Bouchard et al., 2008). Currently, only two measures purport to measure mentalization (operationalised as ‘reflective functioning’) in middle childhood: the Child Reflective Functioning Scale applied to the Child Attachment Interview (Ensink et al., 2013; Shmueli-Goetz et al., 2008), and the Friends and Family Attachment Interview (Bizzi et al., 2022; Steele & Steele, 2005). This paucity of measures has limited the scope of research. It is therefore valuable to review research using both ‘mentalization’ and ‘theory of mind’ measures in middle childhood to aggregate what is known about this under-researched, but potentially significant, developmental period.

This review builds on substantial evidence which suggests that maltreated children in middle childhood show differences in social cognition, including difficulties with cognitive and affective perspective-taking (Burack et al., 2006; Cigala & Mori, 2022; Harwood & Farrar, 2006); emotion perception and recognition (Bérubé et al., 2023; Fishbein et al., 2009); and social understanding (Carpendale & Lewis, 2004; Luke & Banerjee, 2012). Two systematic reviews have addressed related questions: Benarous and colleagues examined evidence of impaired cognitive theory of mind among maltreated school-aged children (Benarous et al., 2015), and Luke and Banerjee reviewed literature on social understanding among maltreated children (Luke & Banerjee, 2013). Neither review included studies exploring the concept of mentalization, and both included samples which extended beyond ages 5–12. Perhaps contrary to expectations, both systematic reviews found ambiguous evidence of links between maltreatment and poorer theory of mind capacity during middle childhood. Benarous and colleagues identified evidence of impaired false-belief understanding among maltreated children, but mixed evidence of hostile attribution and perspective-taking difficulties. Similarly, Luke and Banerjee’s meta-analysis identified an overall negative effect of maltreatment on emotion recognition and understanding but found mixed evidence of effects on perspective-taking and attributional biases.

Building on these past reviews, the present review incorporates evidence from the last decade, includes studies using measures from both mentalization and theory of mind traditions, and focuses on the developmental period of middle childhood. The aim of the present study is to systematically examine whether the literature shows evidence of an association between exposure to childhood maltreatment and mentalization/theory of mind in middle childhood.

## Methods

A systematic literature review was conducted, following the PRISMA guidelines (Page et al., 2021). The protocol for this review was pre-registered on PROSPERO (available at: https://www.crd.york.ac.uk/PROSPERO/view/363819).

### Sources of Information and Search Strategy

Five electronic bibliographic databases were searched for peer-reviewed journal articles (Web of Science Core Collection, Scopus, PsycInfo, MEDLINE, IBSS) and a further three were searched for dissertations/theses (EThOS, OpenAIRE, ProQUEST). All searches were conducted within the date range 1 st January 1980–22nd February 2024, except for EThOS, which could only be searched up until 19th October 2022 due to an ongoing technical outage. The start date reflects that the 1980 s marked the publication of the first works on theory of mind and mentalization (Fonagy & Higgitt, 1990; Wimmer & Perner, 1983). Potentially relevant key words were discussed and agreed among authors, prioritising breadth to avoid overlooking relevant items. Table 1 shows the search terms used for this initial search, divided into the exposure (maltreatment), age range (middle childhood), and outcome variables (mentalization/theory of mind).

**Table 1:** Summary of search terms used to search electronic databases

Main categories (combined with AND)	Synonyms (combined with OR)
Maltreatment	1) Maltreatment 2) Abuse 3) Neglect 4) Trauma 5) Post-traumatic stress 6) Childhood adversity 7) Adverse childhood experience
Middle childhood	8) School-aged 9) Child 10) Middle childhood 11) Adolescent 12) Early adolescence
Theory of mind/Mentalization	13) Theory of mind 14) Mentalization 15) Reflective function 16) False belief 17) Empathy 18) Mind-minded

### Eligibility Criteria

Peer-reviewed journal articles and unpublished dissertations/theses meeting the following a priori criteria were included:

i) Reported exposure to any type of child maltreatment, defined as ‘abuse and neglect that occurs to children under 18 years of age. It includes all types of physical and/or emotional ill-treatment, sexual abuse, neglect, negligence and commercial or other exploitation’ (World Health Organization, 2023). Self-report, parent-report, and administrative records were all considered to be acceptable sources of information about maltreatment. Studies using measures including other adverse childhood experiences (such as parental incarceration or divorce) were eligible for inclusion provided the measure also included maltreatment exposure. Studies of samples at high-risk of maltreatment (such as care-experienced children) were only included if the sample was explicitly described as experiencing maltreatment.
ii) Included no fewer than 15 participants, to exclude statistically underpowered studies (Turner et al., 2013).
iii) Reported on a sample with mean age 5–12 years, even where some participants were outside this age range.
iv) Reported a measure of ‘mentalization’ and/or ‘theory of mind’. To maximise the number of eligible studies, any quantitative measure (including observational, self-report, adult-report, and interview-based instruments) which purported to measure theory of mind or mentalization/reflective function was acceptable, irrespective of evidence of the validity of the measure. Non-standard measures were therefore included where the authors defined these as assessing the variables of interest.
v) Reported a within- or between-groups analysis of the association between maltreatment exposure and mentalization/theory of mind.
vi) Written in English.
vii) Published between 1 st January 1980 and 22nd February 2024.

Observational study designs were included in this review, including cross-sectional, case-control, and cohort studies. Where a longitudinal design was used, baseline or pre-intervention data were extracted where this data was available. Studies using any sampling approach were eligible for inclusion, including random, stratified, convenience, and clinical sampling. Qualitative studies, literature reviews, conference abstracts, posters and letters were excluded. The eligibility of studies was not affected by mental health or neurodevelopmental diagnoses, and there were no other exclusion criteria.

## Study Selection

**Fig. 1:**
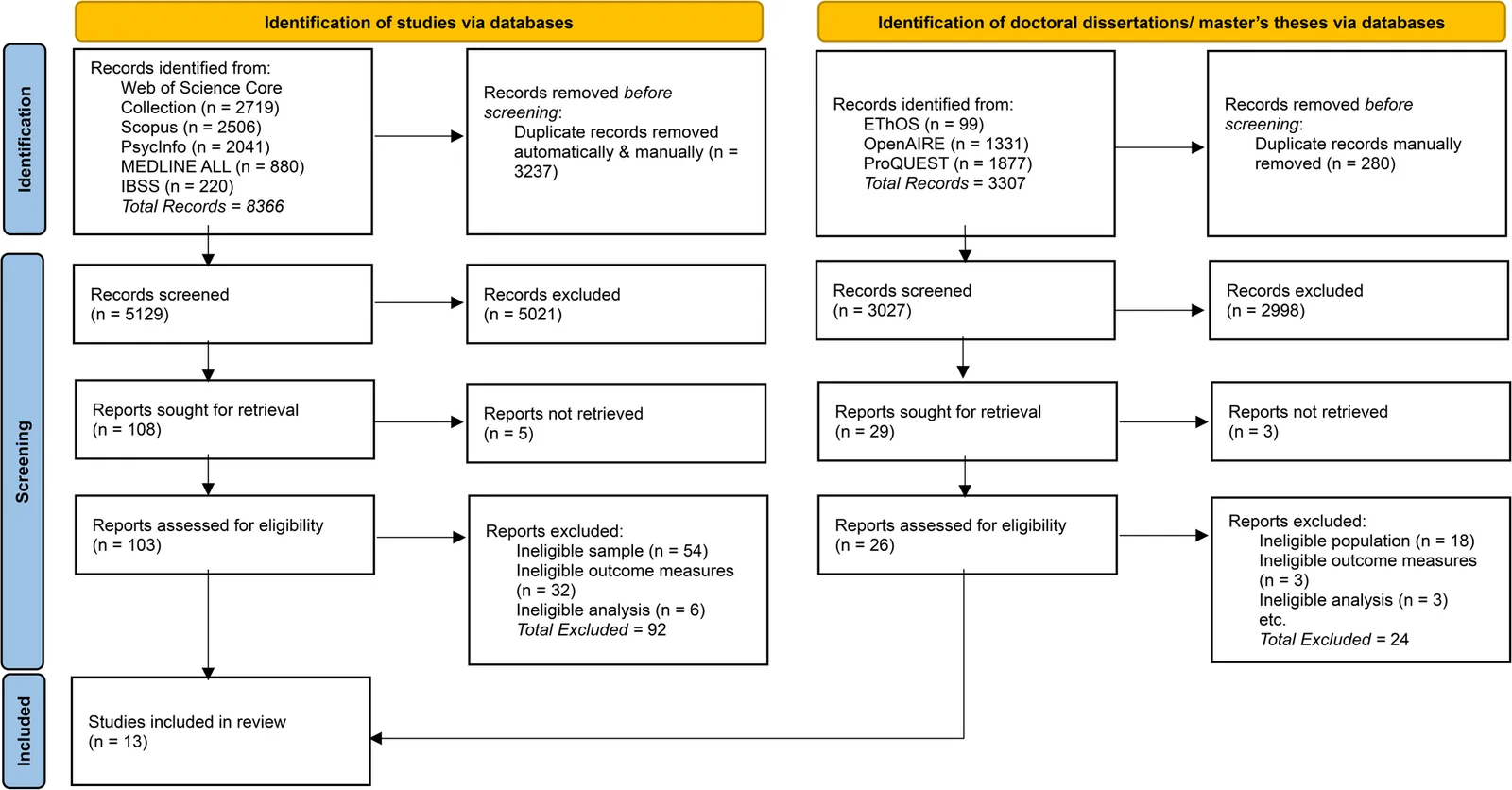
Preferred Reporting Items for Systematic Reviews and Meta-Analyses (PRISMA) flow diagram, showing process of study selection

The full PRISMA flow-chart summarising the search and screening process is presented in Fig. 1. Following the initial search stage, all citations were imported into EndNote x20 (The EndNote Team, 2013). Automatic de-duplication was completed and checked manually by AKD. The initial search yielded 5129 peer-reviewed journal articles and 3027 dissertations/theses for screening following de-duplication. Initial screening of all items to review each title and abstract was completed by AKD (5021 peer-reviewed articles and 2998 dissertations/theses excluded at this stage). 10% of titles and abstracts were additionally reviewed by MW, and interrater reliability was found to be excellent (*Κ* = 0.95). All discrepancies were resolved in discussion between AKD and MW. The remaining articles and dissertations/theses were sought for full-text retrieval and assessed for eligibility by AKD and MW, with excellent interrater reliability (*Κ* = 0.85). Discrepancies were again discussed and agreed in conversation with AKD, MW, SH, and NM. No study authors were contacted as no additional information was required for decisions about study selection to be made.

## Data Extraction

Data from the included studies were extracted by AKD using a standardised coding spreadsheet in Microsoft Excel, with fields pre-registered on PROSPERO. This process was verified by the other authors, with no discrepancies identified. The variables identified included sample characteristics; recruitment source(s); type(s) of maltreatment; measure(s) of maltreatment; outcome variable(s) reported; and task(s) used to assess mentalization. A detailed list of the variables extracted is presented in the Supplementary Materials (Appendix A).

## Study Quality Assessment

Risk of bias for each study was assessed using the Observational Study Quality Evaluation (OSQE) tool (Drukker et al., 2021), using the case-control or cross-sectional versions depending on study design. This tool uses a checklist to evaluate the representatives of the study population, the validity of the independent and dependent variables, and the statistical methods used. How to score each item is pre-defined in detail by the rater before scoring begins (for detail on risk of bias assessment for this review, see the Supplementary Materials [Appendices B & C]). An overall score is generated from 0 to 16 with higher numbers suggesting greater validity of methods and findings. The OSQE emphasises qualitative aspects of quality assessment, so comments about study validity are also incorporated into the narrative synthesis. All papers were coded by AKD and 20% were double-coded by MW. There were no discrepancies in these ratings.

## Results

### Overview

The results of this review are presented as a narrative synthesis. Due to the heterogeneity of study designs and measures used in the included studies, no meta-analysis was possible. A total of 13 studies (11 peer-reviewed journal articles and 2 dissertations/theses) using 12 unique samples were included in the review. Two papers reported on the same data (Ensink et al., 2016, 2017). Sample and study characteristics for each of the included studies are summarised below in Tables 2 and 3. Studies used samples from the USA (4), Canada (4), Turkey (2), Italy (1), Australia (1), and the United Kingdon (1). The included studies used a mixture of case-control and cross-sectional designs, and a variety of methods were used to measure maltreatment and mentalization. Although the present review considers mentalization and theory of mind to be interchangeable terms, the two sections below summarise theory of mind and mentalization studies separately, reflecting the terminology used by study authors. Within each section, sub-sections are organised according to the methods used to assess the outcome variable.

**Table 2:** Summary of study and sample characteristics of included theory of mind studies

Authors (Year)	Country	*N*	% Female	Mean age (range)	Sample recruited from	Control recruited from	Study design	Maltreatment measure	Type of maltreatment	Tasks used	Psychopathology measure
Cicchetti et al. (2003)	USA	203 (M) 315 (NM)	35% (M) 45% (NM)	6.0 (3–8) (M) 6.2 (3–9) (NM)	Child protective services	Neighbourhood flyers, public assistance program	Case-control	MCS	PA/SA/EA/N	Unexpected-contents tasks	N/A
Colvert et al. (2008)	UK	118 (M) 47 (NM)	55% (M) 35% (NM)	11.0 (M) 11.0 (NM)	International adoption program	Adoption agencies	Case-control	Duration of institutional deprivation	N	Strange Stories: Assigning intent to story characters in cartoon story	SCQ & ADI-R; DA; RS
Heleniak and McLaughlin (2020)	USA	121 (PA/SA) 76 (EA) 123 (NM)	46% (PA/SA) 46% (EA) 45% (NM)	12.8 (8–16) (PA/SA) 13.1 (8–16) (EA) 12.4 (8–16) (NM)	Education and community programs	Same as sample (see left)	Case-control and cross-sectional	CECA/VEX-R/CTQ/PTSD-RI	PA/SA/EA	Cartoon narrative completion task: assigning intentions to characters	CBCL YSR; KSADS
Lewis-Morrarty et al. (2012)	USA	37 (M) 24 (NM)	49%	5.0 (4–6)	Clinical intervention trial	Participation in research study	Case-control	Social work reports	N	Penny-hiding game: role-taking measure	N/A
O’Reilly and Peterson (2015)	Australia	52 (M) 53 (NM)	46% (M) 59% (NM)	9.0 (4–13) (M) 8.4 (5–13) (NM)	Child guidance clinics	Education and childcare programs	Case-control	MMCS	PA/SA/EA/N	False belief task; belief-emotion test	N/A
Pashley (2010)	USA	47	45%	9.9 (5–14)	Residential treatment	N/A	Cross-sectional	ACEs	PA/SA/EA/N	Content analysis of narrative therapy sessions	Medical records

Abbreviations: *ACEs*, Adverse Childhood Experiences questionnaire (Dube et al., 2003; Felitti et al., 1998); *ADI-R*, Autism Diagnostic Interview revised (Rutter, Le Couteur et al., 2003); *CBCL YSR*, Youth Self Report Child Behavior Checklist (Ebesutani et al., 2011); *CECA*, Child Experiences of Care and Abuse Interview (Bifulco et al., 1994); *CTQ*, Childhood Trauma Questionnaire (Bernstein et al., 1997); *DA*, Disinhibited attachment based on parental interview; *EA*, Emotional abuse; *KSADS*, Kiddie Schedule for Affective Disorders and Schizophrenia (Kaufman et al., 1997); *M*, Maltreated; *MCS*, Maltreatment Classification System (Barnett et al., 1993); *MMCS*, Modified Maltreatment Classification System (English & the LONGSCAN Investigators, 1997); *N*, Neglect; *N/A*, Not applicable; *NM*, Non-maltreated; *PA*, Physical abuse; *PTSD-RI*, UCLA PTSD Reaction Index (Steinberg et al., 2004); *SA*, sexual abuse; *SCQ*, Social Communication Questionnaire (Rutter, Bailey et al., 2003); *VEX-R*, Violence Exposure Scale for Children – Revised (Fox & Leavitt, 1995)

**Table 3:** Summary of study and sample characteristics of included mentalization studies

Authors (Year)	Country	*N*	% Female	M age (range)	Sample recruited from	Control recruited from	Study design	Maltreatment measure	Type of maltreatment	Tasks used	Psychopathology measure
Ensink et al. (2015)	Canada	46 (M) 48 (NM)	74% (M) 60% (NM)	9.6 (M) 14.9 (NM)	Child protective services	Education and health services	Case-control	Clinical case history	SA	CAI-CRFS	N/A
Ensink et al. (2016)	Canada	74 (M) 94 (NM)	59% (M) 55% (NM)	9.4 (7–12) (M) 9.3 (7–12) (NM)	Child protective services and health services	Education and health services	Case-control	Medical, social work, and police reports	SA	CAI-CRFS	CDI; CBCL
Ensink et al. (2017)	Canada	74 (M) 94 (NM)	59% (M) 55% (NM)	9.4 (7–12) (M) 9.3 (7–12) (NM)	Child protective services and health services	Education and health services	Case-control	Medical, social work, and police reports	SA	CAI-CRFS	CDI; CDC; CBCL; CSBI
Halfon et al. (2020)	Turkey	100	38%	7.0 (4–10)	Child psychotherapy clinic	N/A	Correlational	ACEs	PA/EA	Attachment Doll Story Completion Task	CBCL
Tessier et al. (2016)	Canada	39 (M) 32 (NM)	77% (M) 57% (NM)	9.5 (M) 9.0 (NM)	Child protective services and health services	Education and daycare programs	Longitudinal case-control	Clinical case history	SA	CAI-CRFS	N/A
Ünal (2019)	Turkey	108	61%	7.1 (3–10)	Child psychotherapy clinic	N/A	Cross-sectional	ACEs	PA/SA/EA/N	Attachment Doll Story Completion Task	CBCL
Zaccagnino et al. (2015)	Italy	24 (M) 35 (NM)	50% (M) 49% (NM)	11.3 (10–13) (M) 10.7 (9–13) (NM)	Foster care	School	Case-control	Social work reports	PA/SA/EA/N	CAI-CRFS	N/A

Abbreviations: *ACEs*, Adverse Childhood Experiences questionnaire (Dube et al., 2003; Felitti et al., 1998); *CAI-CRFS*, Child Attachment Interview: Child Reflective Functioning Scale (Ensink et al., 2013); *CBCL*, Child Behavior Checklist (Achenbach & Rescorla, 2001); *CDC*, Child Dissociative Checklist (Putnam et al., 1993); *CDI*, Child Depression Inventory (Kovacs, 1985); *CSBI*, Child Sexual Behavior Inventory (Friedrich et al., 2001), *EA*, Emotional abuse; *M*, Maltreated; *N*, Neglect; *N/A*, Not applicable; *NM*, Non-maltreated; *PA*, Physical abuse; *SA*, sexual abuse

### Mentalization Studies

Seven studies explored the relationship between maltreatment exposure and mentalization (Ensink et al., 2015, 2016, 2017; Halfon et al., 2020; Tessier et al., 2016; Ünal, 2019; Zaccagnino et al., 2015). Five studies used a primarily case-control design, and two used a cross-sectional design (Halfon et al., 2020; Ünal, 2019). Generally, the included studies found that maltreatment exposure was associated with poorer mentalization, but one paper found no significant associations between mentalization and maltreatment exposure (Ünal, 2019).

### Child Reflective Functioning Scale

Five of the included studies, comprising four unique samples, assessed mentalization by using the Child Reflective Functioning Scale (CRFS, Ensink et al., 2013) to code the Child Attachment Interview (CAI, Shmueli-Goetz et al., 2008). This manualised age-specific measure focuses on assessing mentalization (reflective functioning) based on children’s narratives about themselves, others, and their interpersonal relationships (Bizzi et al., 2022). The CRFS produces separate scores for self-focused reflective functioning and other-focused reflective functioning, which can be combined into a global reflective functioning score.

Studies which used the CRFS consistently found that children exposed to maltreatment had poorer mentalizing scores than children without maltreatment exposure (Ensink et al., 2015, 2016, 2017; Tessier et al., 2016; Zaccagnino et al., 2015). For instance, Ensink and colleagues (2015) found that sexually abused children had significantly lower global, self-focused, and other-focused reflective functioning scores compared to the non-abused control group (respectively *t*(93) = 4.51, *p*<.001; *t*(93) = 3.84, *p*<.001; and *t*(93) = 4.44, *p*<.001). A similar finding reported by Ensink and colleagues in two further papers (Ensink et al., 2016, 2017) showed that children with experience of sexual abuse had a significantly lower mean level of global reflective functioning (*F* = 23.40, *p*<.01), and results from a regression analysis showed that exposure to sexual abuse had a direct effect on mentalization (*β* = − 0.518, *p*<.01). These findings also converge with Tessier and colleagues’ finding that self- and other-focused reflective functioning were both significantly lower among sexually abused children than non-abused controls (*t*(58) = 2.20, *p*<.05 and *t*(58) = 2.63, *p*<.05). This finding remained significant after controlling for age and socio-economic status. Each of these papers focused exclusively on sexually abused children, and only one study using the CRFS sampled children exposed to other types of maltreatment (Zaccagnino et al., 2015). This study found that CRFS scores were significantly higher among children who were not living in residential care due to abuse and neglect compared to those who were (*F(*1,57) = 61.24, *p*≤.00). These studies therefore consistently suggest that maltreatment may negatively affect children’s mentalization.

### Mental State Talk

Two studies used a different coding system – the Coding System for Mental State Talk (Bekar et al., 2014) – to code a doll-based story completion task (Bretherton et al., 1990) with a focus on talk about mental states used as a proxy measure of mentalization (Halfon et al., 2020; Ünal, 2019). Although both studies explored correlations between maltreatment exposure and mental state talk (MST), they produced mixed findings. Ünal (2019) did not identify any significant associations between maltreatment exposure and emotional MST. However, Halfon and colleagues (2020) found that there was a significant association between emotional neglect and impaired MST, even when controlling for sex and expressive language ability (*r* =.28, *p* <.05). Positive associations were also found between emotional neglect and imbalanced other/self MST (*r* =.25, *p* <.05), physical abuse and imbalanced cognitive/emotional MST (*r* =.27, *p* <.05), and physical abuse and imbalanced action/emotional MST (*r* =.23, *p* <.05). These studies therefore showed a complex and mixed set of findings when using MST to assess the relationship between maltreatment and mentalization.

### Theory of Mind Studies

Six studies explored the relationship between maltreatment exposure and theory of mind (Cicchetti et al., 2003; Colvert et al., 2008; Heleniak & McLaughlin, 2020; Lewis-Morrarty et al., 2012; O’Reilly & Peterson, 2015; Pashley, 2010). Five of these studies used a primarily case-control design, of which one also incorporated a correlational analysis (Heleniak & McLaughlin, 2020), and the other study used a cross-sectional design (Pashley, 2010). All of the included studies found some evidence that higher levels of maltreatment exposure were associated with poorer ratings of theory of mind.

### False-Belief Tasks

Two studies primarily used a false-belief paradigm to assess theory of mind (Cicchetti et al., 2003; O’Reilly & Peterson, 2015). In a well-controlled study using a variant of Perner, Leekam, and Wimmer’s (1987) unexpected contents “smarties” task and Gopnik and Astington’s (1988) false-belief book task, Cicchetti and colleagues (2003) found that significantly fewer maltreated children (56.3%) demonstrated false-belief understanding than non-maltreated children, both from low (71.3%, *χ2*(1) = 15.23, *p*<.025) and middle (78.1%, *χ2*(1) = 14.78, *p*<.001) socio-economic groups. Hierarchical regression showed that maltreatment status remained a significant predictor of false-belief understanding after controlling for age and socio-economic status (*β* = −0.70, *p*<.05).

Using a similar methodology, O’Reilly and Peterson (2015) investigated the effect of maltreatment on advanced theory of mind using a range of theory of mind tasks, including two false-belief tasks – the changed location “Sally-Ann” task (Baron-Cohen et al., 1985) and an unexpected contents task (Wellman & Liu, 2004). To assess affective aspects of theory of mind, a belief-emotion test was used – asking how a doll would feel when receiving something with unexpected contents (Wellman & Liu, 2004). In addition to these first-order theory of mind tasks, second-order theory of mind was assessed using a variant of the false-belief “window” task (Hughes et al., 2000). The full theory of mind scale of tasks developed by Wellman and Liu (2004) was also administered, which assesses four theory of mind concepts in addition to false belief. Consistent with Cicchetti and colleagues, this study found further evidence that maltreated children performed significantly more poorly on the false-belief tasks than non-maltreated children (*t*(103) = 2.13, *p=*.035).

However, on the overall theory of mind scale, there was no significant difference between the non-maltreated and maltreated groups, although a sub-sample of maltreated children aged 7.5 years or younger did show significantly poorer performance compared to the non-maltreated group (*t*(36) = 2.89, *p*<.01). The second-order false belief task also showed no significant difference between groups, although the advanced belief-emotion task within the theory of mind scale did indicate poorer performance among maltreated children (*χ2*(2) = 27.87, *N* = 105, *p*<.001).

Both studies also reported additional analyses of the impact of severity and nature of abuse on theory of mind. O’Reilly and Peterson (2015) identified a negative correlation between maltreatment severity and theory of mind false-belief task performance, and Cicchetti and colleagues (2003) found the effect of maltreatment on theory of mind was strongest among children whose maltreatment began earlier in life. They also reported that both physical abuse and emotional maltreatment had significant effects on false belief performance when considered individually, whereas sexual abuse and neglect did not predict differences in false-belief performance, possibly due to the relatively small number of participants who had experienced sexual abuse.

### Narrative Tasks

Two studies used narrative tasks to assess advanced theory of mind (Colvert et al., 2008; Heleniak & McLaughlin, 2020). Using the Strange Stories task to assess children’s ability to assign intent to story characters in a vignette (Happé, 1994), Colvert and colleagues (2008) found that there were significant differences in theory of mind performance between adoptees from within the UK, international non-institutionalised adoptees, and severely neglected institutionalised international adoptees (*I*(2, 179) = 6.77, *p*<.01). Although the specific analysis is not reported, the authors state that post-hoc analysis showed that international institutionalised adoptees performed significantly more poorly on theory of mind than the other adoptee groups, and the degree of deficit was associated with length of time in conditions of neglect and deprivation (*r*=-.37, *p*<.001).

Unlike Colvert and colleagues, Heleniak and McLaughlin (2020) assessed both cognitive and affective aspects of theory of mind using a story completion task with forced-choice response options (Sebastian et al., 2012). This study found that cognitive theory of mind accuracy was lower among children with experience of physical or sexual abuse (*F*(1, 245) = 5.38, *p*=.021) and emotional abuse (*F*(1, 245) = 7.71, *p*=.006) than among children who had not been exposed to these types of maltreatment. Correlational analysis additionally found that cognitive theory of mind accuracy was negatively associated with physical or sexual abuse (*r*=-.13, *p*<.05), emotional abuse (*r=*-.15, *p*<.05), and violence frequency (*r*=-.17, *p*<.01), but not other maltreatment types. This study found that children who had experienced emotional abuse scored significantly lower on affective theory of mind than non-emotionally abused children (*F*(1, 245) = 9.84, *p=*.002), but there were no significant differences in affective theory of mind scores between other maltreatment types. Both studies assessing theory of mind with narrative tasks therefore showed some significant associations between maltreatment and cognitive and affective theory of mind performance, but this finding was not consistent across all maltreatment types.

### Non-Standard Tasks

In two studies, theory of mind was assessed using non-standard methods (Lewis-Morrarty et al., 2012; Pashley, 2010). Lewis-Morrarty and colleagues (2012) used a penny-hiding game, in which the child is asked to imitate a researcher who hides a penny in one hand behind their back and presents both hands for a partner to guess which hand the penny is in (Oswald & Ollendick, 1989). This task was originally designed to assess for role-taking and social competence but is presented by Lewis-Morrarty as a measure of theory of mind. The imitative nature of this task led to very high average scores (mean 7.9 points out of 9, with standard deviation 2.0). However, in a comparison between maltreated children in foster care and non-maltreated children not in foster care, the maltreated group received significantly lower scores on the theory of mind measure when controlling for receptive language scores.

Unlike other theory of mind studies, Pashley (2010) reported on a clinical sample who had received exposure-based narrative therapy. Transcripts of sessions during which children were asked to describe their adverse experiences of care were coded using the Narrative Content Analysis System (Foa et al., 1995), with a focus on congruent mental state terms and correct mental state attributions. This measure is presented by the authors as a theory of mind variable. A simple correlational analysis found that adverse childhood experiences (including maltreatment exposure) were significantly negatively associated with the composite theory of mind variable (*r*=-.48, *p*<.01). Unlike the other included studies, these two studies did not use methods with reported evidence of validity, but both reported results consistent with the hypothesis that maltreatment exposure is negatively associated with mentalization in middle childhood.

### Critical Appraisal

The critical appraisal of each included paper, using the OSQE for either case-control or correlational designs, is presented in the Supplementary Materials (Appendix B). The included papers ranged from 6 to 13 ‘stars’ on the OSQE, out of a possible 16 for case-control and 10 for correlational papers. Three questions were not clearly applicable to the papers in this review: no papers attempted to analyse modifiers (Q15), there was no evidence presented about whether control groups had a history of poor mentalization/theory of mind (Q7), and there were no explicit references to pre-registered protocols or planning of analyses (Q14). The papers which scored lowest on the OSQE tended to use non-valid approaches to assess the key variables of maltreatment (Q3) and theory of mind/mentalization (Q6) and did not use appropriate sampling methods (Q1 and Q2). The breadth of approaches to assessing maltreatment also tended to vary between cases and controls (Q5), which undermined the validity of several papers.

## Discussion

This systematic review examined research exploring whether there is an association between maltreatment exposure and mentalization among children in middle childhood. Thirteen studies were included in the review. Overall, the present review provides indicative evidence, consistent with related reviews, that higher levels of exposure to maltreatment may be associated with poorer mentalization capacity in middle childhood (Benarous et al., 2015; Luke & Banerjee, 2013). However, there was some mixed evidence from papers which specifically assessed advanced and affective theory of mind, and one paper reported no significant association between mentalization and maltreatment exposure. These findings are discussed in the context of evidence across childhood development, and plausible explanations for the observed association are proposed. Finally, the quality of the body of literature, along with the strengths and weaknesses of the present review are considered, before reflecting on implications for future research, policy, and practice.

The results of this review provide insight into the effects of maltreatment on the understudied period of middle childhood. Previous studies have identified deficits in mentalization linked to maltreatment among younger children (Egeland & Sroufe, 1981; Pears & Fisher, 2005; Valentino et al., 2011) and adolescents and adults (Crawford et al., 2022; Simon et al., 2019; Taubner et al., 2016), and the present review found evidence replicating this association in middle childhood. In this regard, the findings of this review support the hypothesis that maltreatment may have consistent effects on the development of mentalization across childhood (Allen, 2019; Cicchetti & Olsen, 1990). Poor mentalization among maltreated children in middle childhood may be linked to distinctive social, emotional, and behavioural presentations, so middle childhood merits exploration as a distinct stage of mentalizing development (Ensink & Mayes, 2010).

### Maltreatment and Complex Mentalizing in Middle Childhood

Surprisingly, the present review did not find consistent evidence that more elaborate forms of mentalizing captured by ‘advanced’ theory of mind measures, which normatively develop during middle childhood, were significantly impaired among maltreated children (Heleniak & McLaughlin, 2020; O’Reilly & Peterson, 2015). This negative finding may partly be explained by the rich social context of middle childhood, as new networks of peer and adult relationships begin to form in the school environment (Feiring & Lewis, 1991). In this setting, the mentalization skills of maltreated children may rapidly develop as complex social cognition is increasingly required to navigate everyday social experiences. This developmental catch-up may be further facilitated by experiences of non-maltreating adults at school or in out-of-home care who can provide attuned and mentalizing relationships (Joseph et al., 2014). This would further explain the age effect detected by O’Reilly and Peterson (2015), whereby younger children aged less than 7.5 years (who may not have had as much opportunity for reparative relational experiences) showed poorer overall theory of mind scores than age-matched non-maltreated children, but older maltreated children did not. It is therefore plausible that maltreatment in early childhood has different impacts across middle childhood depending partly on the social experiences of children within that period.

This review also found mixed evidence of the effect of maltreatment in middle childhood on performance during affectively-focused theory of mind tasks. Although O’Reilly and Peterson (2015) found that performance on the belief-emotion task was significantly poorer among maltreated children, Heleniak and McLaughlin (2020) found that the only type of maltreatment associated with poorer affective theory of mind performance was emotional abuse, and no other forms of maltreatment appeared to be related to affective theory of mind capacity. This finding partially diverges from the results of the meta-analysis by Luke and Banerjee (2013), which identified a significant negative effect of maltreatment on emotion recognition and understanding but did not report differing effects by type of maltreatment. One explanation for this discrepancy may lie in the nuanced evidence about the effects of maltreatment on emotion understanding. Whilst several papers indicate that maltreated children have overall inhibited capacities to recognise emotions (Bérubé et al., 2023; Fishbein et al., 2009), some evidence suggests that maltreated young people may be *better* at recognising certain emotions, such as anger and fear, which may confound generic measures of emotion perception (Shackman & Pollak, 2005). It is therefore possible that affective theory of mind tasks may capture several sub-capacities which are, variably, either impaired or enhanced by maltreatment.

Studies using the well-validated CRFS assessment of mentalization did consistently find poorer global mentalization among sexually abused school-aged children. The overall CRFS score incorporates affective and cognitive as well as self-and other-focused aspects of mentalization and consequently may be less influenced by the complex effect of maltreatment on affective mentalizing discussed above. It is equally plausible that the CRFS is more sensitive to the aspects of affective mentalization affected by maltreatment than affective theory of mind tasks. However, this effect would need to be confirmed by evidence from an independent research team not involved in the development of the measure (Bizzi et al., 2022).

### Possible Causal Mechanisms

Whilst the studies reviewed indicate an association, they do not establish the mechanisms by which early maltreatment may lead to poor mentalization/theory of mind in childhood. Current research has begun to examine potential mediating and moderating variables which may influence the observed relationship between maltreatment and social cognition, including individual, family-level, and broader contextual factors (Colley et al., 2024).

From a cognitive perspective, it is possible that the findings simply reflect that multiple adverse experiences inhibit domain-general processes, impacting capacities which may include mentalization (Ehlert, 2013). As chronic exposure to stressful or traumatic events in childhood can have a dysregulating effect on several neurobiological systems (Cross et al., 2017), poor mentalization may be only one aspect of a cascade of developmental impacts rooted in physiological changes (Hughes et al., 2017; Maercker et al., 2022).

Co-occurring adversities, such as parental substance use, parental mental health difficulties, and lower socio-economic status may have independent and confounding effects on social cognition, alongside maltreatment (Evans & France, 2022; Lamb et al., 1997; Nilsson et al., 2012). Few of the included studies in this review controlled for other potentially impactful adverse or traumatic experiences besides maltreatment, so the findings could be plausibly explained by this generic mechanism (Walsh et al., 2019).

However, experiences of maltreatment, compared to other forms of trauma and adversity, may have distinct effects on mentalization. Some research suggests that interpersonal traumas such as maltreatment have different psychological effects compared to other forms of adversity, leading to higher rates of post-traumatic stress disorder and more fear-based symptoms (Forbes et al., 2012; Hughesdon et al., 2021). Moreover, the present review identified some evidence of differentiated effects between types of maltreatment experiences: for example, Heleniak and McLaughlin (2020) identified that emotional abuse, but not physical or sexual abuse, was associated with impaired affective theory of mind. These differentiated impacts suggest that the association between different types of maltreatment and mentalization may require a more refined explanation, accounting for the specifically relational context of maltreatment.

An alternative explanation which links the interpersonal context of maltreatment to difficulties with mentalizing in childhood is offered by the theory of epistemic trust. This recent development to the mentalization-based framework proposes that attuned caregiving produces the capacity for child mentalization via appropriately calibrating the infant’s capacity to trust in socially-communicated knowledge (‘epistemic trust’) (Fonagy et al. 2015, 2017a). Experiences of mentalizing caregiving are theorised to relax the infant’s natural stance of epistemic vigilance – caution about the validity of socially-communicated information – enabling the infant to learn about the world, and themselves, from the mind of their caregiver.

The failure to develop epistemic trust has been proposed as a way of understanding the difficulties with mentalization observed in maltreated children. Epistemic trust theory proposes that maltreating caregivers tend to fail to mentalize their child (Luyten et al., 2017; Rosso, 2022), missing interactive cues or responding to these cues in frightening and unpredictable ways (Fonagy et al., 2007; Luyten et al., 2020). The child’s epistemic vigilance therefore remains fixed, with no mechanism to relax this mistrusting stance (Luyten & Fonagy, 2019; Sperber et al., 2010). Children who are chronically ‘frozen’ in epistemic mistrust – a state described as ‘epistemic hypervigilance’ or ‘epistemic petrification’ – are hypothesised to be unable to integrate representations about the minds of others, undermining the capacity to mentalize (Fonagy et al., 2015; Fonagy & Allison, 2014). In this sense, children who experience maltreatment may perceive their caregiver’s mind as being hostile and unsafe, disabling their emerging capacity to learn about the world or themselves from the mind of the caregiver. Consequently, maltreated children may be delayed or inhibited in mentalizing as others’ minds – beyond their caregiver’s – may also be perceived as dangerous or unhelpful (Knox, 2016). Some studies have begun to explore relationships between maltreatment, epistemic trust, and mentalization in adolescents and adults childhood (Campbell et al., 2021; Driehuis, 2021; Kampling et al., 2022; Schröder-Pfeifer et al., 2022). This recent vein of research has led to a preliminary examination of epistemic trust as an explanatory model for the dynamics of foster care relationships with maltreatment children (Sprecher et al., 2022), but further empirical examination of the links between maltreatment, epistemic trust, and mentalization are required.

#### Quality of Literature

The findings of this review are somewhat weakened due to methodological issues in the included papers. These weaknesses were reflected in the critical appraisal, which identified that most papers were of either moderate or poor quality. Across the 13 included studies, mentalization was assessed using eight distinct methods, each building on slightly different conceptualisations of the outcome variable. Although the reliability of first-order false-belief tasks is acceptable (Hughes et al., 2000; Wellman et al., 2001), advanced theory of mind measures show poor consistency across different assessment approaches, to the extent that different tasks may be construed as measuring distinct underlying constructs (Bloom & German, 2000; Hayward & Homer, 2017; Osterhaus & Bosacki, 2022). The included papers used several non-standard methods to assess mentalization/theory of mind, reducing the comparability of results across studies. Although studies using the CRFS showed more consistent results, most of these studies were authored by the team which developed the measure, which may have introduced publication bias (Ensink et al., 2013, 2015).

Moreover, a considerable breadth of approaches were used to assess maltreatment exposure among their samples. Several papers did not assess for maltreatment exposure among community controls, and maltreatment was often assessed using potentially unreliable information sources such as administrative records. Few studies included data on the characteristics, chronicity, or severity of maltreatment exposure, and sample size limitations precluded more detailed analysis of maltreatment subtypes in several studies (Manly et al., 1994).

#### Directions for Future Research

Although the findings of this review show a consistent association, the mixed quality of evidence prevents any strong conclusions from being drawn. Further research, including studies that examine causal mechanisms, is therefore required to test the hypothesis that maltreatment inhibits mentalization in middle childhood (Fonagy & Bateman, 2016). Well-controlled longitudinal prospective studies with large samples and repeated, reliable, and valid measures of mentalization, psychopathology, and other related factors such as language development would be beneficial to establish the stability of the impacts of maltreatment over time. This research might additionally consider gene-environment interactions which influence mentalization development (Quevedo et al., 2022; Skuse & Gallagher, 2011; Skyberg et al., 2022). These research designs would also enable exploration of pressing questions about the relationship between mentalizing in middle childhood and adult outcomes including mental health (Chiesa & Fonagy, 2014; Dvir et al., 2014).

Further research may also explore the relationships between the timing, severity, chronicity, and types of maltreatment and their respective impacts on mentalization (Manly et al., 1994). Possible gender differences in the effect of maltreatment on mentalization should also be explored (Gallo et al., 2018). Research could also consider how experiences of therapeutic out-of-home care may impact the developmental catch-up of mentalization among maltreated children (Redfern et al., 2018). Likewise, future research may empirically explore how mechanisms such as epistemic mistrust may mediate the link between maltreatment and mentalization development in middle childhood (Kampling et al., 2022).

Finally, research may move beyond nomothetic approaches examining population-level associations towards idiographic and qualitative explorations of how maltreated young people experience relationships with others (Atiqul Haque et al., 2017; Pigeon et al., 2023; Wilson et al., 2023). This would also provide insight into the significance of mentalization from the perspective of young people with experiences of maltreatment (Foster, 2017). Qualitative research would also be able to accommodate complexity in the experiences of young people across their different experiences of relationships (Jaffrani et al., 2020; Oaksford & Frude, 2004; Sprecher et al., 2021).

#### Implications for Practice

Mentalization difficulties may underly some of the presenting clinical difficulties of maltreated children, and may therefore represent a meaningful target for clinical interventions such as Mentalization-Based Treatment for Children (MBT-C) (Midgley et al., 2021; Midgley & Vrouva, 2013), which has also been adapted for children in middle childhood who have experienced early adversity (Midgley et al., 2018). Practitioners should receive training assess mentalizing difficulties, and these difficulties should hold weight as part of clinical formulations (Muller & Midgley, 2015). Maltreated children may struggle to establish epistemic trust with clinicians and, whatever modality of therapy is offered, one of the tasks of therapy may be to ‘open’ the inhibited mentalizing capacity of the young person within the context of a secure therapeutic relationship (Fonagy & Allison, 2014; Jaffrani et al., 2020; Li et al., 2022; Midgley et al., 2018). In this respect, the provision of psychological support for maltreated children may be essential to enable recovery from the relational harm of maltreatment.

### Strengths and Limitations

This systematic review has several strengths. To the authors’ knowledge, it is the first review to systematically address the literature about maltreatment and mentalization/theory of mind in middle childhood, contributing to the field of research about social cognition and maltreatment. The findings of this review suggest a trend of significant results that indicate higher maltreatment exposure is associated with poorer mentalization. This provides a clear basis for further research to build upon this finding.

One limitation of the present review was that a meta-analysis could not be conducted due to the heterogeneity of methodologies in the included papers, meaning that an overall pooled effect size estimate was not calculated. It is not possible to infer the direction of the observed effects due to the exclusively cross-sectional research designs used. Moreover, no papers reported moderating or mediating variables, so this review does not capture evidence of how and why maltreatment is associated with poorer mentalization. The definition of maltreatment used by the present review may have also incorporated overly diverse experiences, although this reflects the wider quality of the evidence base about child maltreatment.

An additional limitation was that the review used two complex concepts – mentalization and theory of mind – which have been defined in a variety of ways (Duschinsky & Foster, 2021). These ‘umbrella’ concepts are closely related to a number of concepts not explicitly included in the review, including empathy, mindfulness, psychological mindedness, perspective-taking, emotion recognition, insight, and social understanding (Choi-Kain & Gunderson, 2008; Luyten et al., 2020). Likewise, by focusing on the mentalization-based theoretical framework, this review excluded other theoretical perspectives which may contribute to explaining the effects of maltreatment on social cognition. Although these concepts were beyond the purview of the present review, a more comprehensive examination of associations between maltreatment and these related concepts may further enrich our understanding of how maltreatment affects children’s social cognition.

## Conclusion

The present review found indicative evidence that maltreatment exposure may be associated with poorer mentalization/theory of mind in middle childhood. Although most evidence supported this trend, the quality of the evidence base is significantly limited due to the breadth of measurement approaches used and some poorly designed studies. This evidence may therefore only be taken as provisional, and further research is needed to explore how individual profiles of maltreatment may affect specific aspects of mentalization/theory of mind across childhood development.

## Supplementary Information

Below is the link to the electronic supplementary material.

Supplementary Material 1 (DOCX 25.1 KB) 

## Data Availability

The data that support the findings of this study are available from the corresponding author, AKD, upon reasonable request.
