# Supplementary material for: A Systematic Review of the Relationship Between Maltreatment and Mentalization in Middle Childhood
Source: J Child Adolesc Trauma. 2026 Jun 6;19(4):2037–55. doi: 10.1007/s40653-026-00924-0 (PMC13737262; doi:10.1007/s40653-026-00924-0)
Supplement: Supplementary file 1 [file 40653_2026_924_MOESM1_ESM.docx]

**Supplementary Materials**

***Appendix A***

*Details of variables extracted.*

Data were extracted from each paper in relation to the following variables:

- Authorship details:
  - Authors
  - Year of publication
- Study setting
  - Geographic location
  - Recruitment source(s)
- Sample characteristics
  - Sample size
  - Participant demographics (age and gender)
  - Reported mental health or neurodevelopmental diagnoses
  - Type(s) of maltreatment exposure in sample
- Methodological characteristics
  - Study design
  - Measure(s) of maltreatment
  - Task(s) to assess outcome variables
- Summary of results
  - Reported associations

|  |  | Case-Control OSQE | | | | | | | | | |  | Correlational OSQE | | |
| --- | --- | --- | --- | --- | --- | --- | --- | --- | --- | --- | --- | --- | --- | --- | --- |
|  |  | Cicchetti (2003) | Colvert (2008) | Ensink (2015) | Ensink (2016) | Ensink (2017) | Heleniak (2020) | Lewis-Morrarty (2020) | O'Reilly (2015) | Tessier (2016) | Zaccagnino (2015) |  | Halfon (2020) | Pashley (2010) | Ünal (2019) |
|  | Q1 | Y | N | N | N | N | Y | N | N | N | N |  | N | Y | N |
|  | Q2 | Y | N | Y | Y | Y | Y | N | Y | Y | Y |  | N/A | N/A | N/A |
|  | Q3 | Y | N | Y | Y | Y | Y | N | Y | Y | N |  | Y | Y | Y |
|  | Q4 | Y | Y | N | N | N | Y | N | Y | N | N |  | Y | Y | Y |
|  | Q5 | N | N | N | N | N | Y | N | N | N | N |  | N/A | N/A | N/A |
|  | Q6 | Y | Y | Y | Y | Y | Y | N | Y | Y | Y |  | N | N | N |
|  | Q7 | N | N | N | N | N | N | N | N | N | N |  | N/A | N/A | N/A |
|  | Q8 | Y | Y | N | N | N | Y | Y | Y | Y | Y |  | N/A | N/A | N/A |
|  | Q9 | N | N | N | N | N | N | N | N | N | N |  | N/A | N/A | N/A |
|  | Q10 | N | N | N | N | N | Y | Y | N | N | N |  | N/A | N/A | N/A |
|  | Q11 | Y | Y | Y | Y | Y | Y | Y | Y | Y | Y |  | Y | Y | Y |
|  | Q12 | N | N | N | N | N | N | N | N | N | N |  | N | N | N |
|  | Q13 | Y | Y | Y | Y | Y | Y | Y | Y | Y | N |  | Y | N | Y |
|  | Q14 | N | N | N | N | N | N | N | N | N | N |  | N | N | N |
|  | Q15 | N | N | N | N | N | N | N | N | N | N |  | N | N | N |
|  | Q16 | Y | Y | Y | Y | Y | Y | N | Y | Y | Y |  | Y | Y | Y |
| TOTAL | | 11 | 8 | 8 | 8 | 8 | 13 | 6 | 10 | 9 | 7 |  | 6 | 6 | 6 |

***Appendix B***

*Critical appraisal of each included study using OSQE assessment.*

***Appendix C***

*OSQE questions and scoring guidance.*

Q1	Is the sample optimal for both internal validity and representativeness?

*To score this item, the balance between appropriate population homogeneity and external validity was considered. To gain a star, the sample of maltreated children needed to be reasonably random.*

Q2	Is the data collected in one population or are cases and controls selected in different populations?

*To score this item, ‘one population’ was considered to mean one age group within the same area, even if recruitment strategies targeted maltreated and non-maltreated children by different means.*

Q3	Is the assessment of the main independent variable valid?

*To score this item, the validity of maltreatment measurement was considered. To gain a star, a reliable method of measuring maltreatment had to be used. Papers which alluded to maltreatment without measuring it did not gain a star.*

Q4	Is the presence of the independent variable optimal? (i.e. severity of exposure, compliance, fidelity to intervention, presence of risk factor, discontinuation of exposure)

*To score this item, the focus was on how thoroughly exposure was reported. Therefore, to gain a star, papers needed to report the extent, onset, and nature of maltreatment where possible. Given that there is no ‘optimal’ degree of exposure to maltreatment, the severity of exposure to maltreatment within the sample was not considered relevant when scoring this item.*

Q5	Is the method of assessment independent variable the same in cases and controls?

*To score this item, the method of assessing maltreatment was considered. Only studies which used the same method to assess maltreatment in maltreated and non-maltreated samples gained a star for this item.*

Q6	Is assessment of the dependent variable valid? (In other words: Is case definition adequate?)

*To score this item, the validity of the assessment of theory of mind/ mentalization was considered. To gain a star, studies needed to use a standard, statistically validated method of assessment.*

Q7	Do controls have a history of the disease?

*To score this item, we considered whether non-maltreated samples had a history of difficulties with mentalization or theory of mind. Studies could only gain a star if they reported on this.*

Q8	Is follow-up sufficiently long to assess the outcome?

*To score this item, the time between maltreatment exposure and assessment of theory of mind/ mentalization were considered. Where the timing of maltreatment was not reported, a star could not be given.*

Q9	Does non-response likely introduce bias?

*To score this item, any reported information about non-response was considered. This answer is reverse-coded, so a ‘No’ is given a star. Papers which did not report on non-response were assessed according to whether bias was likely as a result of the research design.*

Q10	Is non-response similar in cases and controls?

*Where non-response rates were not reported, a ‘No’ answer was recorded.*

Q11	Did the authors use methods to properly deal with missing data? (including loss to follow-up)

*To score this item, reported processes for dealing with missing data are considered. Where no missing data were reported, studies were given a star for this item.*

Q12	Is there a conflict of interest? (e.g. funding or connections with pharmacological industry)

*To score this item, any reported conflicts of interest were considered. This answer is reverse-coded, so a ‘No’ is given a star.*

Q13	Does the statistical analysis control for the relevant confounders? Also takes into account matching.

*To score this item, efforts to control any relevant confounders were considered. Where confounders were controlled via matching, studies were also given a star.*

Q14	Did the reporting of the results follow a protocol? In other words, were only a priory intended analyses reported? As opposed to cherry picking.

*To score this item, any reported use of a protocol for analysis and reporting were considered. To gain a star, studies had to describe the planned analyses, ideally with pre-registration.*

Q15	Are effect modifiers analysed, correctly?

*To score this item, relevant effect modifiers were considered. Generally, this was not a relevant item for the nature of studies in the present review.*

Q16	Is sample size sufficient, looking at calculations/explanation provided by the authors?

*To score this item, reported information about the sufficiency of the sample size were considered. Studies including analyses which accounted for the sample size or equivalent power analyses were given a star.*
